# Invasive Pneumococcal Disease in Tuscany Region, Italy, 2016–2017: Integrating Multiple Data Sources to Investigate Underreporting

**DOI:** 10.3390/ijerph17207581

**Published:** 2020-10-19

**Authors:** Filippo Quattrone, Gabriele Donzelli, Sara D’Arienzo, Marco Fornili, Francesco Innocenti, Silvia Forni, Laura Baglietto, Lara Tavoschi, Pier Luigi Lopalco

**Affiliations:** 1 Department of Translational Research and New Surgical and Medical Technologies, University of Pisa, 56126 Pisa, Italy; filippo.quattrone@med.unipi.it (F.Q.); gabriele.donzelli@for.unipi.it (G.D.); pierluigi.lopalco@unipi.it (P.L.L.); 2 Tuscany Regional Health Agency (Agenzia Regionale di Sanità della Toscana), 50141 Florence, Italy; sara.darienzo@ars.toscana.it (S.D.); francesco.innocenti@ars.toscana.it (F.I.); silvia.forni@ars.toscana.it (S.F.); 3 Department of Clinical and Experimental Medicine, University of Pisa, 56126 Pisa, Italy; marco.fornili@gmail.com (M.F.); laura.baglietto@unipi.it (L.B.)

**Keywords:** invasive pneumococcal disease, surveillance systems evaluation, vaccine-preventable diseases, capture–recapture analysis

## Abstract

Invasive pneumococcal disease (IPD) is a vaccine-preventable disease characterized by the presence of *Streptococcus pneumoniae* in normally sterile sites. Since 2007, Italy has implemented an IPD national surveillance system (IPD-NSS). This system suffers from high rates of underreporting. To estimate the level of underreporting of IPD in 2016–2017 in Tuscany (Italy), we integrated data from IPD-NSS and two other regional data sources, i.e., Tuscany regional microbiological surveillance (Microbiological Surveillance and Antibiotic Resistance in Tuscany, SMART) and hospitalization discharge records (HDRs). We collected (1) notifications to IPD-NSS, (2) SMART records positive for *S. pneumoniae* from normally sterile sites, and (3) hospitalization records with IPD-related International Classification of Diseases, Ninth Revision, Clinical Modification (ICD9) codes in discharge diagnoses. We performed data linkage of the three sources to obtain a combined surveillance system (CSS). Using the CSS, we calculated the completeness of the three sources and performed a three-source log-linear capture–recapture analysis to estimate total IPD underreporting. In total, 127 IPD cases were identified from IPD-NSS, 320 were identified from SMART, and 658 were identified from HDRs. After data linkage, a total of 904 unique cases were detected. The average yearly CSS notification rate was 12.1/100,000 inhabitants. Completeness was 14.0% for IPD-NSS, 35.4% for SMART, and 72.8% for HDRs. The capture–recapture analysis suggested a total estimate of 3419 cases of IPD (95% confidence interval (CI): 1364–5474), corresponding to an underreporting rate of 73.7% (95% CI: 34.0–83.6) for CSS. This study shows substantial underreporting in the Tuscany IPD surveillance system. Integration of available data sources may be a useful approach to complement notification-based surveillance and provide decision-makers with better information to plan effective control strategies against IPD.

## 1. Introduction

Invasive pneumococcal disease (IPD) is a life-threatening disease characterized by the isolation of *Streptococcus pneumoniae* or detection of its nucleic acid or antigen from a normally sterile site [1]. The most common clinical presentations are meningitis, sepsis, or bacteremic pneumonia with a case fatality ratio ranging from 10–25% among affected patients [2].

In 2017, 23,886 confirmed cases of IPD (6.2 cases per 100,000 inhabitants) were reported to the European Surveillance System (TESSy) by 29 European countries [3]. The groups more affected were children under 1 year of age (14.5 cases per 100,000 population) and adults aged ≥65 years (18.9 cases per 100,000 population) [3].

Vaccination against *S. pneumoniae* is the most effective public health measure for preventing IPD both among vaccine recipients (direct effect) and among unimmunized populations (indirect “herd” effect) [4]. However, the development of a universal vaccine against *S. pneumoniae* is challenged by its high genetic diversity. At least 98 different serotypes of *S. pneumoniae* have been identified [5], although the 10 most common serotypes (8, 3, 22F, 19A, 12F, 9N, 15A, 10A, 11A, and 23B) account for 66% of typed isolates in Europe [3]. Two types of pneumococcal vaccine are now in use: pneumococcal conjugate vaccine (PCV, available in 10- or 13-valent formulations) and pneumococcal polysaccharide vaccine (PPV, working against 23 serotypes). Conjugate antigens stimulate a more effective humoral immune response than polysaccharide-only antigens, particularly in young children [5]. Introduction of PCV in 2000 resulted in a remarkable decline in IPD in countries where universal pneumococcal vaccination was implemented [6]. Immune pressure from widespread use of PCVs, however, resulted in increasing prevalence of nonvaccine serotypes (serotype replacement) [5]. The multidrug-resistant serotype 19A emerged worldwide as a predominant serotype after the introduction of the initial 7-valent formulation of pneumococcal conjugate vaccine (PCV7) [5,7]. In Italy, universal free-of-charge pneumococcal vaccination was introduced in 2012 in children; regions autonomously choose which vaccine to purchase, in the context of the duopoly between 10-valent and 13-valent anti-pneumococcal vaccine. The switch of some European countries or regions from PCV13 to PCV10 (including the Italian region of Piedmont) has led to intense debate [6,8,9]. According to a recent World Health Organization publication, local epidemiological data, alongside vaccine supply and economic factors, should inform local vaccination strategies [10]. In this scenario, an effective surveillance system for IPD is essential to understand local epidemiology, serotype distribution, and antibiotic resistance rates and to monitor the impact of vaccinations [11].

IPD surveillance at the European level was initiated in 2010 with a progressively increasing number of countries introducing it at the national level over the past decade [12]. However, a large heterogeneity exists among countries with respect to the surveillance system in place and the adopted case definition [3].

In 2007, Italy developed an IPD national surveillance system (IPD-NSS) as part of a wider national passive surveillance system for invasive bacterial diseases caused by *S. pneumoniae*, *Neisseria meningitidis*, and *Haemophilus influenzae* [12]. The Italian National Institute of Health (Istituto Superiore di Sanità, ISS) coordinates IPD-NSS with the financial support of the Italian Ministry of Health. In IPD-NSS, cases are reported to the surveillance database [13] by hospitals or regional health authorities on a voluntary basis [14]. In 2017, 1705 IPD cases were notified to the IPD-NSS (2.4 cases per 100,000 inhabitants) [3]. A high variability in rates of reported cases and serotyping of clinical isolates is present among the 21 Italian Regions [15]. Tuscany, a region in central Italy with about 3.7 million of inhabitants, reported 74 cases in 2016 and 53 in 2017 with a notification rate of 2.0 cases per 100,000 inhabitants in 2016 and 1.4 in 2017. This finding is below the European mean incidence of 5.4 cases per 100,000 in 2016 and 6.2 in 2017 and divergent from previous studies that reported higher levels of hospitalization for IPD in Tuscany [16]. These observations have pointed toward the need for an assessment of the quality of the IPD surveillance system currently in place in Tuscany [15,17].

The aim of this study was to evaluate the quality of epidemiological information on IPD in Tuscany during 2016–2017, integrating data from IPD-NSS with two other regional data sources, i.e., the Tuscany regional microbiological surveillance system (called SMART, Microbiological Surveillance and Antibiotic Resistance in Tuscany) and the regional hospitalization discharge records (HDRs) via record linkage and capture–recapture analysis.

## 2. Materials and Methods

### 2.1. Description of Data Sources

**Figure 1:**
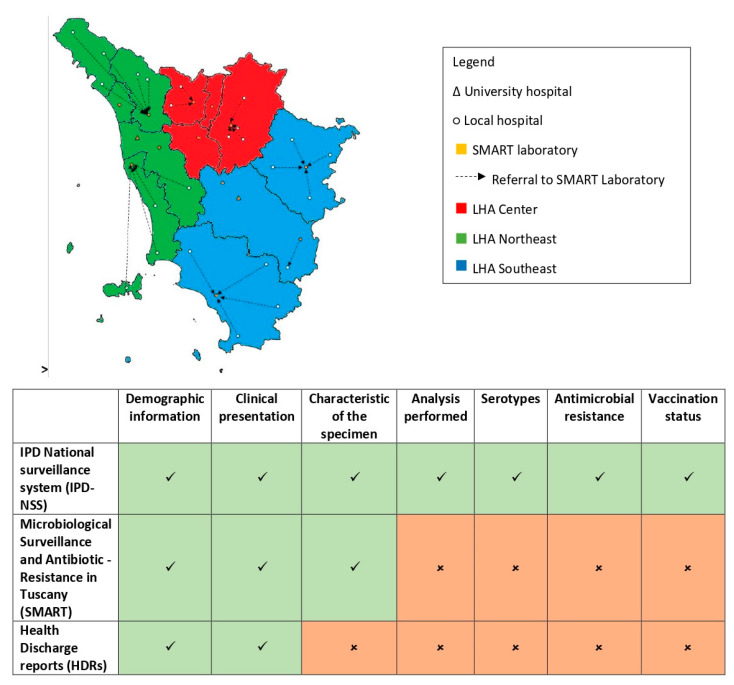
The Tuscany invasive pneumococcal disease data sources. Different data sources can be used to estimate invasive pneumococcal disease (IPD) burden in Tuscany. All doctors can notify a case to the national IPD Surveillance system, while all laboratories of the microbiological surveillance system SMART (Microbiological Surveillance and Antibiotic Resistance in Tuscany) collect specimens from all hospitals and communicate positive results to the regional health authority (ARS, Agenzia Regionale di Sanità Toscana). Hospital discharge records are also collected by the ARS and accessible for research. Different features of IPD are covered by different data sources.

We extracted data on IPD cases in Tuscany in the period 2016–2017 from three different sources: IPD-NSS, SMART, and HDRs. Inclusion criteria and collected variables for the different sources are shown in Figure 1.

(1) IPD-NSS. The IPD-NSS adopts the definition of IPD issued by the European Commission [1]. All cases of IPD reported in Tuscany in 2016 and 2017 were included. The variables collected were patient identification number, demographic characteristics (age, sex), clinical presentation of IPD, vaccination status, healthcare service/hospital identifier, characteristics of collected sample (date of collection, type of sample, analysis performed), and serotype. According to IPD-NSS protocol [13], cases of IPD should be notified to the local Public Hygiene Service within 12 h by doctors, microbiologists, or hospital health directors via a paper report form. The Public Hygiene Service uploads the data onto a national web-based platform. Data are also transmitted to the regional health information system. At the same time, the isolated strain is sent to the regional or national reference laboratory for confirmation and serotype characterization.

(2) SMART. In 2013, Tuscany set up a regional microbiological surveillance system. The system collects data of microbiological cultures from 14 laboratories, covering the entire region. ARS (Agenzia Regionale di Sanità Toscana, Health Regional Agency) gathers data from different laboratory information systems and controls their quality. For the present analysis, we included cases of samples (blood, liquor, or other normally sterile sites) testing positive for *S. pneumoniae* in 2016 and 2017. The variables collected were regional universal identifier (IDUNI), calculated from patients’ Italian fiscal code, or a laboratory-specific identifier, demographic information (sex and age or date of birth), place of sample collection, characteristics of collected sample (date of collection, type of sample), and antibiotics susceptibility profile.

(3) HDRs. All discharge records from hospitalizations in Tuscany are digitalized and transmitted from hospitals to a regional archive for administrative purposes. At the regional level, HDRs are anonymized with the IDUNI identifier. The HDR database is available to the ARS for research purposes. We considered all patients resident in Tuscany in 2016–2017 with at least one HDR generated in Tuscany in that period that contained a primary or secondary diagnosis of streptococcal septicemia (International Classification of Diseases, Ninth Revision, Clinical Modification (ICD9)-CM038.0), pneumococcal septicemia (ICD9-CM038.2), pneumococcal meningitis (ICD9-CM320.1), streptococcal meningitis (ICD9-CM320.2), pneumococcal peritonitis (ICD9-CM567.1), or pneumococcal infection in conditions classified elsewhere (ICD9-CM041.2). In case of multiple hospitalizations with IPD-related HDRs for the same patient over the study period, data were extracted from the first one. HDRs included IDUNI, demographic details of patient (sex and age or date of birth), place and period of hospitalization, and diagnoses at discharge coded according to the International Classification of Diseases, Ninth Revision, Clinical Modification (ICD9-CM).

### 2.2. Identification of Common Cases among Sources

The records of the three data sources were linked via the IDUNI identifier. When IDUNI was not available, records were linked on the basis of the patient’s date of birth or age, sex, and place and period of hospitalization. If linkage was possible for all available variables, records were associated. The total number of cases was calculated, identifying overlapping among different databases. Unique cases were then collected in a common database, the combined surveillance system (CSS), which included the variables common to all databases (sex, age, and place of collection of samples or place of hospitalization) [18].

### 2.3. Description of Surveillance Systems, Calculation of Incidence, and Estimation of Underreporting

We performed a descriptive analysis on the three source databases and CSS using Stata/SE, version 14 (StataCorp LP, College Station, TX, USA)

We calculated the incidence of IPD per 100,000 inhabitants at the level of the three regional local health authorities (LHAs, in Tuscany: Northwest, Center, Southeast). We summed cases from community and university hospitals insisting on the same LHA and divided them by the number of inhabitants of LHAs resident on 31 December 2017 (source: ARS). Completeness of each data source was calculated by dividing the number of cases reported by the total number of cases resulting from data linkage (CSS estimate).

### 2.4. Capture–Recapture Analysis

Capture–recapture analysis is a well-established method to estimate the quality of reporting systems, and it was previously applied to IPD and other invasive bacterial diseases [18,19,20,21,22,23,24].

A log-linear capture–recapture method was developed to estimate the number of cases not captured by any of the three sources. The application of the method was based on the assumption of a closed population (0.05% decrease in the period 2016–2017 [15]). To account for the possible dependence among sources, we introduced into the model the two-way interactions between sources. To account for potential heterogeneous catchability, we adjusted the model for (1) year (2016, 2017), (2) place of origin (university hospitals, LHA Northwest, LHA Center, LHA Southeast), (3) age (≤24, 25–64, ≥65 years), and (4) sex (male, female). On the basis of the selected model, we estimated the total number of cases and the corresponding 95% asymptotic confidence intervals (CIs) overall and by year, place of origin, sex, and age group. For these analyses, we used the *glm* and *step* functions and package *emmeans* (estimated marginal means for the total number cases, including reported and unreported) of R, version 3.5.2. According to definition given by the European Centre for Disease Prevention and Control (ECDC) [25], underreporting was defined as “the failure to adequately report symptomatic cases that have sought medical advice” and calculated as the ratio of cases not reported in CSS and the number of cases estimated by the capture–recapture analysis.

This study complies with the Declaration of Helsinki and with Personal Data Protection Code (Legislative decree no. 196/2003 of 30 June 2003) on the protection of personal data. Informed consent and approval from the local ethics committee were unnecessary because the information involved is routinely recorded for surveillance purposes and treated in anonymous form.

## 3. Results

In the period 2016–2017, 127 cases of IPD were notified to the IPD-NSS, including 70 (55.1%) males and 57 (44.9%) females. Information on age, sex, and isolation were specified in all records. Meningitis was the most frequent clinical presentation of IPD (73 episodes, 57.5%), followed by bacteremic pneumonia (69 episodes, 54.3%) and septicemia (61 episodes, 48.0%). *S. pneumoniae* was isolated from cerebral fluid in 71 cases (55.9%), from blood in 55 cases (43.3%), and from another sterile site in one case (0.8%). The diagnostic analyses were performed using culture methods in 82 cases (64.6%), PCR in 42 cases (33.1%), antigen detection in 29 cases (22.8%), and direct microscopic exams in 13 cases (10.2%). Of the 64 (50.4%) cases with available information on serotype, 42 (33.1%) were caused by any PPV23 serotype, 16 (12.6%) were caused by any PCV13 serotype, and eight (6.3%) were caused by a PCV10 serotype, while 22 (17.3%) cases were caused by a serotype not included in any PCV vaccine. Of the 84 (66.1%) patients whose vaccination status was reported, five were vaccinated (6.0%).

During the same period, in SMART, 320 cases of IPD were reported, including 177 (55.3%) males and 143 (44.7%) females. The unique anonymous identifier was correctly specified for 250 cases (78.1%), while age, sex, and data of isolation were specified in 294 (91.9%) cases. All cases had at least one of the factors used to link data. *S. pneumoniae* was isolated from cerebral fluid in 264 cases (82.5%), blood in 110 cases (34.4%), and another sterile site in nine case (2.8%). The results for the antimicrobial resistance tests on the 42 characterized *S. pneumoniae* isolates showed that 23 (7.2%) isolates were resistant to erythromycin, whereas 15 (4.7%) were resistant to penicillin, and only four (1.3%) were cefotaxime/ceftriaxone-resistant.

Finally, during 2016–2017, 658 IPD cases were found in HDRs, including 359 (54.6%) males and 299 (45.4%) females. The unique anonymous identifier was correctly specified for 588 cases (89.4%), while age, sex, and admission and discharge data were always reported. On the basis of the case definition, 385 (58.5%) cases of IPD were retrieved from HDRs with a diagnosis of streptococcal septicemia (ICD9-CM 038.0), 136 (20.7%) involved pneumococcal septicemia (ICD9-CM 038.2), 124 (18.8%) involved pneumococcal meningitis (ICD9-CM 320.1), 25 (3.8%) involved streptococcal meningitis (ICD9-CM 320.2), 18 (2.7%) involved pneumococcal infection in conditions classified elsewhere (ICD9-CM 041.2), and one (0.2%) involved pneumococcal peritonitis (ICD9-CM 567.1).

### 3.1. The Combined Surveillance System (CSS)

After data linkage, we identified 904 cases recorded in at least one data source and included them in CSS. Record linkage was performed through IDUNI or, if missing, with age, sex, and period of isolation. Performance levels of data linkage between SMART and HDRs were similar with both techniques; considering cases in both data sources with correct and incorrect IDUNI, the percentage of linked cases was respectively 11.7% and 6.4%. Moreover, with reference to 88 cases correctly matched with IDUNI, 82 (93.2%) would have also been correctly matched on the basis of non-IDUNI fields.

**Figure 2:**
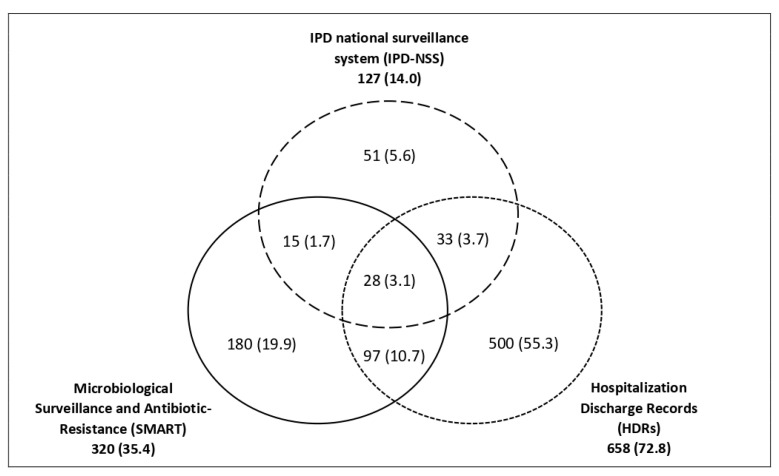
Venn diagram showing the number (percentage) of cases of invasive pneumococcal disease identified from three sources: the IPD national surveillance system (IPD-NSS), the Microbiological Surveillance and Antibiotic Resistance in Tuscany (SMART), and the regional hospitalization discharge records (HDRs) from 2016 to 2017. In total, 904 single cases are individuated by the three combined systems.

Only 3.1% (28/904) of cases were listed in all three data sources (Figure 2). Of all cases, 280 (31.0%) were notified by LHA Center, 268 (29.6%) by LHA Northwest, 142 (15.7%) were notified by LHA Southeast, 102 (11.3%) were notified by University Hospital Careggi, 59 (6.5%) were notified by University Hospital of Pisa, 26 (2.9%) were notified by University Hospital of Siena, and 22 (2.4%) were notified by University Hospital Meyer. For four (0.4%) cases, the information was not available (see Table S1, Supplementary Materials).

Table 1 shows the number of cases in 2016 and 2017 for each of the three sources and the CSS, including sex, age, and notification rate. Male-to-female ratio varied among the sources with a slight predominance of male subjects. Patients aged more than 65 years old were the most represented age class (63.9%). Notification rates varied among areas and years with a lower notification rate in the southeast area. The average 2016–2017 CSS notification rate was 12/100,000 inhabitants. Completeness was 14.0% for IPD-NSS, 35.4% for microbiological surveillance, and 72.8% for HDRs.

### 3.2. Capture–Recapture Estimates

The results of the capture–recapture log-linear analysis are shown in Table 2. The estimated total number of IPD cases was 3419 (95% CI: 1364–5474), with an underreporting rate for the CSS of 73.7% (95% CI: 34.0–83.6). The selected model included the main effects and the two-way interactions between sources and the interactions of each source with year, age, and sex, as well as the interactions of year and place of origin with both age and sex. On average, higher underreporting was found in 2016 and at older ages. Similar underreporting was found for males and females; with respect to place of origin, reporting was higher for the Northwest LHA. The estimated 2016–2017 average incidence of IPD was 45.6/100,000 inhabitants (95% CI: 18.2–57.8).

## 4. Discussion

To our knowledge, this is the first attempt to assess the sensitivity and performance of IPD surveillance in an Italian region, namely, Tuscany, by combining multiple available data sources. Our study indicates a substantial level of incompleteness in all three data sources assessed, with HDRs as the best performing source. Our capture–recapture analysis highlighted a valuable number of cases underreported by all the considered sources.

IPD-NSS in Italy suffers from a significant regional variability with regions in the north of Italy systematically notifying more cases than regions in the south [15]. However, according to a recent analysis [26], notified cases in Italy have shown a sustained increase over the last decade, with the number of reported IPD cases exceeding HDR cases identifiable through ICD9 codes. Yet, compared to national data or regional data from northern Italy, Tuscany shows a consistently lower IPD notification rate [27]. In Tuscany, the process of notification is still largely not digitalized, and the awareness of healthcare workers on the importance of infectious disease notification is still low. Even when cases are reported, suboptimal completeness of relevant information hampers appropriate monitoring. Serotype characterization was available for 50% of reported cases, and patients’ vaccination status was largely not collected. Yet, such information would be useful to assess the impact of universal pneumococcal vaccination rolled out in the country in 2012 and to inform vaccine strategies, including vaccine product selection [10], at the regional level, as procurement of medical products is in the remit of the regional health authority [6,8].

Despite the microbiological surveillance SMART gathering data from all regional hospital laboratories, a substantial level of underreporting was observed in our study. Loss of information due to incomplete data collection and suboptimal integration with the regional IDUNI system by some laboratories of the network could be identified among the possible root causes of SMART incompleteness [28], alongside intrinsic limitation of the diagnostic methods. While essential for the confirmation of the diagnosis, microbiological cultures suffer from a high level of false negatives both in adult patients with a septic status and in children with symptoms compatible with IPD [29,30]. Previous studies showed low sensitivity of the laboratory-based case definitions and, subsequently, the IPD burden was considerably underestimated when based solely on bacterial cultures on blood or cerebrospinal fluid [31,32]. In particular, one study exploring the incidence of non-laboratory confirmed IPD found it was 1.1–2.8-fold higher than that based on culture-confirmed or probable invasive pneumococcal disease [31]. This result is comparable with the number of cases identified in HDRs being twofold higher than that reported in SMART.

The integration of epidemiological, microbiological, and clinical sources via data linkage and capture–recapture analysis is a well-established method to assess the completeness of surveillance of IPD [19,20,21] and other infection diseases at a regional or national level in Italy and elsewhere [18,23,33,34,35]. Using data linkage, we estimated a mean annual incidence rate for IPD in Tuscany during the review period substantially higher (12.1/100,000 vs. 1.7/100,000) than that resulting from the sole IPD-NSS. Our estimates are comparable with those obtained from other IPD surveillance systems. In particular, the CSS-derived IPD incidence estimate was comparable to those obtained from better performing surveillance systems in other Italian regions (e.g., in 2017, 8.7/100,000 IPD cases in Trentino-Alto Adige, 7.1/100,000 in Piedmont [15]) or other European countries (e.g., in 2017, 15.9/100,000 cases in Slovenia or 13.2/100,000 in the Netherlands [3]). Epidemiology of IPD is not expected to be different across Italy, in consideration of similar vaccination coverage levels and healthcare system structures. In Italy, over the past decade, an increasing number of IPD cases reported through the IPD-NSS has been noted [26], suggesting an ongoing improvement of IPD cases reporting procedures and highlighting opportunities for further progress.

When considering the capture–recapture analysis, the estimated incidence raised to 45.6/100,000 IPD cases per year, with a certain degree of geographical variations within the region, highlighting a higher level of under reporting for older age groups. Our estimate was comparable to that of a Belgian study applying different models of capture–recapture (44 to 58/100,000 IPD cases in 2010 in >65 years old) [20]. The observed higher level of underreporting among older subjects has already been described for IPD [20,21] and may be linked to a lower level of hospitalization in this population group or, in the case of severe illness in the elderly, to the tendency not to give a specific diagnosis. Furthermore, a geographical variability in IPD underreporting has been described in the literature [20,22], correlated to the distance between laboratories and hospitals [20]. This observation is compatible with the findings from this study, as a higher level of underreporting was described in the Southeast LHA, characterized by a larger area and lower population density as compared to other regional LHAs. However, detailed information concerning diagnostic, medical, and reporting practices is difficult to determine from routinely collected data; therefore, the specific causes for the observed intraregional differences are challenging to infer and will require further field investigations.

This study has some limitations. Our selection of HDRs cases was based on a set of ICD9 codes explicitly linked to a pneumococcal infection in a normally sterile site. This approach could have led either to an underestimation of cases (e.g., bacteremic pneumonia classified as pneumococcal pneumonia) or to an overestimation (diagnosis based on unjustified clinical suspicion or coding mistakes). We also used conservative data linkage rules to link the IPD-NSS and the records without IDUNI to the other sources, which could have led to an incomplete linkage of this source with the others and, consequently, an overestimation of the level of underreported IPD cases in capture–recapture analysis.

The capture–recapture technique has intrinsic limitations and is sensitive to the assumption of homogeneous capture probabilities and source independence, which are rarely respected in epidemiological sources [36]. However, the log-linear parametric method offers the opportunity to account for the heterogeneity and dependence between sources [20]. Our results should be interpreted as a preliminary estimate of the (in)completeness of the CSS and be a stimulus for better epidemiological surveillance of IPD in Tuscany.

## 5. Conclusions

With our study, we provide evidence of substantial underreporting in IPD surveillance in Tuscany with important implications for public health. In particular, the observed incompleteness of IPD-NSS hampers the possibility to effectively monitor the impact of the current vaccine strategy and of pneumococcal antimicrobial resistance in Tuscany. We believe that increasing awareness of the importance of infectious diseases surveillance among healthcare workers and simplifying the case reporting process via complete digitalization and integration with other regional data sources are essential to improve IPD prevention and control. Available technical solutions, as suggested elsewhere [19], such as automatic reminders for clinicians or automatic case reporting integrated in a laboratory information system, could improve the completeness of the current surveillance system.

Our results suggest that systematic integration of IPD surveillance data information with data sources collected for other purposes is an effective tool to assess the level of IPD underreporting and to estimate true disease incidence, to better inform the design and evaluation of public health interventions against IPD.

## Figures and Tables

**Table 1:** Number of cases observed in 2016 and 2017 for each of the three sources and the combined surveillance system (CSS), including sex, age, and origin information.

		IPD-NSS		SMART		HDRs		CSS	
		2016	2017	2016	2017	2016	2017	2016	2017
	**Total**	74	53	136	184	322	336	441	463
**Sex**	Male	46	24	85	92	187	172	260	228
	Female	28	29	51	92	135	164	181	235
**Age (years)**	<1	0	1	1	1	7	5	8	6
	1–4	2	0	4	3	11	5	11	6
	5–14	0	0	1	5	3	6	3	8
	15–24	0	0	3	2	8	3	9	4
	25–44	8	8	15	19	26	30	41	42
	45–64	23	14	33	36	74	63	103	85
	≥65	41	30	79	118	193	224	266	312
**Notification rate** **(*n*/100,000 inhabitants)**	Northwest LHA (835,760 inhabitants)	1.44	2.51	4.07	6.82	14.00	17.59	17.35	21.90
	Center LHA (1,627,964 inhabitants)	3.01	1.41	5.16	6.20	7.99	7.99	12.90	11.92
	Southeast LHA (1,278,713 inhabitants)	1.02	0.70	1.41	2.03	5.87	4.61	6.73	6.41
	All regions (3,742,437 inhabitants)	2.0	1.4	3.6	4.9	8.6	9.0	11.8	12.3

**Table 2:** Estimated number of unreported cases of invasive pneumococcal disease in the CSS according to a log-linear capture–recapture model. CI, confidence interval.

	Observed in CSS	Estimated (95% CI)	Underreporting Percentage (95% CI)
**Total**	900 *	3419 (1364–5474)	73.7 (34.0–83.6)
**Year**			
2016	441	1899 (681–3116)	76.8 (35.2–85.8)
2017	459	1520 (617–2424)	69.8 (25.6–81.1)
**Place of origin**			
University hospitals	209	841 (279–1403)	75.1 (25.1–85.1)
Center	280	1117 (391–1842)	74.9 (28.4–84.8)
Northwest	269	863 (329–1398)	68.8 (18.2–80.8)
Southeast	142	598 (161–1036)	76.3 (11.8–86.3)
**Sex**			
Male	487	1881 (738–3024)	74.1 (34.0–83.9)
Female	413	1538 (613–2464)	73.1 (32.6–83.2)
**Age**			
<25 years	55	102 (39–166)	46.1 (41.0–66.9)
25–65 years	271	888 (324–1435)	69.5 (16.4–81.1)
>65 years	574	2428 (901–3956)	76.4 (36.3–85.5)

* Four cases with missing place of origin were excluded from the capture–recapture analysis.

## Supplementary Materials

The following are available online at https://www.mdpi.com/1660-4601/17/20/7581/s1, Table S1. Integration of three data sources on IPD in a combined surveillance system.
