# Supplementary material for: Invasive Pneumococcal Disease in Tuscany Region, Italy, 2016–2017: Integrating Multiple Data Sources to Investigate Underreporting
Source: Int J Environ Res Public Health. 2020 Oct 19;17(20):7581. doi: 10.3390/ijerph17207581 (PMC7589942; doi:10.3390/ijerph17207581)
Supplement: Supplementary file 1 [file ijerph-17-07581-s001.pdf]

Annex 1. Integration of three data sources on IPD in a combined surveillance system. All percentages are on the total number of cases. Data were reported on the CSS column when a variable was present in at least two sources.

	**IPD-NSS** *IPD National Surveillance System*		**SMART** *Microbiological Surveillance and Antibiotic - Resistance in Tuscany*		**HDRs** *Hospitalization discharge records*		**CSS** *Combined surveillance system*	
**Year**	**2016**	**2017**	**2016**	**2017**	**2016**	**2017**	**2016**	**2017**
**Total (n)**	74	53	136	184	322	336	441	463
**Sex (n, %)**								
Male	46 (62.2)	24 (45.3)	85 (62.5)	92 (50.0)	187 (58.1)	172 (51.2)	260 (59.0)	228 (49.2)
Female	28 (37.8)	29 (54.7)	51 (37.5)	92 (50.0)	135 (41.9)	164 (48.8)	181 (41.0)	235 (50.8)
**Age (n, %)**								
<1 y	0	1 (1.9)	1 (0.7)	1 (0.5)	7 (2.2)	5 (1.5)	8 (1.8)	6 (1.3)
1-4 y	2 (2.7)	0	4 (2.9)	3 (1.6)	11 (3.4)	5 (1.5)	11 (2.5)	6 (1.3)
5-14 y	0	0	1 (0.7)	5 (2.7)	3 (0.9)	6 (1.8)	3 (0.7)	8 (1.7)
15-24 y	0	0	3 (2.2)	2 (1.1)	8 (2.5)	3 (0.9)	9 (2.0)	4 (0.9)
25-44 y	8 (10.8)	8 (15.1)	15 (11.0)	19 (10.3)	26 (8.1)	30 (8.9)	41 (9.3)	42 (9.1)
45-64 y	23 (31.1)	14 (26.4)	33 (24.3)	36 (19.6)	74 (23.0)	63 (18.8)	103 (23.4)	85 (18.4)
≥ 65 y	41 (55.4)	30 (56.6)	79 (58.1)	118 (64.1)	193 (59.9)	224 (66.7)	266 (60.3)	312 (67.4)
**Age (mean)**								
Male > 4 y	65.2	64	64.2	66.2	64.7	67.2	65.4	67.4
Female > 4 y	65.7	64.2	65.9	65.8	71	69.6	68.9	69.5

Pediatric cases (0-4 y)	1.5	0	1.4	2.0	1.4	1.3	1.4	1.3
All	63.7	62.9	62.5	64.6	63.6	66.4	63.9	66.7
**Place of origin (n, %)**								
LHA Northwest	12 (16.2)	17 (32.1)	25 (18.4)	43 (23.4)	102 (31.7)	124 (36.9)	121 (27.4)	148 (32.0)
University Hospital of Pisa	0	4 (7.5)	9 (6.6)	14 (7.6)	15 (4.7)	23 (6.8)	24 (5.4)	35 (7.6)
LHA Center	30 (40.5)	12 (22.6)	65 (47.8)	65 (35.3)	87 (27.0)	91 (27.1)	145 (32.9)	135 (29.2)
University Hospital Careggi	17 (23.0)	11 (20.8)	16 (11.8)	33 (17.9)	28 (8.7)	33 (9.8)	50 (11.3)	52 (11.2)
University Hospital Meyer	2 (2.7)	0	3 (2.2)	3 (1.6)	15 (4.7)	6 (1.8)	15 (3.4)	7 (1.5)
LHA Southeast	13 (17.6)	5 (9.4)	18 (13.2)	18 (9.8)	65 (20.2)	48 (14.3)	76 (17.2)	66 (14.3)
University Hospital of Siena	0	4 (7.5)	0	8 (4.3)	10 (3.1)	11 (3.3)	10 (2.3)	16 (3.5)
**Clinical presentation (n, %)**								
Septicemia	39 (52.7)	22 (41.5)	NA	NA	NA	NA		
Bacteraemic pneumonia	28 (37.8)	41 (24.5)	NA	NA	NA	NA		
Meningitis	33 (44.6)	40 (75.5)	NA	NA	NA	NA		
Other	0 (0)	0 (0)	NA	NA	NA	NA		
**Vaccination status (n, %)**								
Vaccinated	3 (4.1)	2 (3.8)	NA	NA	NA	NA		
Not vaccinated	43 (58.1)	36 (67.9)	NA	NA	NA	NA		
Unknown	28 (37.8)	15 (28.3)	NA	NA	NA	NA		

**Characteristics of sample (n, %)**								
Blood	42 (56.8)	13 (24.5)	113 (83.1)	151 (82.1)	NA	NA	142 (75.1)	156 (72.5)
Liquor	32 (42.2)	39 (73.6)	18 (13.2)	29 (15.7)	NA	NA	42 (22.2)	55 (25.6)
Other	0 (0)	1 (1.9)	5 (3.7)	4 (2.2)	NA	NA	5 (2.7)	4 (1.9)
**Analysis performed (n, %)**								
Direct microscopic exam	4 (5.4)	9 (17.0)	NA	NA	NA	NA		
Culture	52 (70.2)	30 (56.5)	NA	NA	NA	NA		
Antigen detection	16 (21.6)	13 (24.5)	NA	NA	NA	NA		
PCR	20 (27.0)	22 (41.5)	NA	NA	NA	NA		
**Serotyping (n, %)**								
PPV23 serotypes	39 (52.7)	3 (5.7)	NA	NA	NA	NA		
PCV10 serotypes	7 (9.5)	1 (1.9)	NA	NA	NA	NA		
PCV13 serotypes	14 (18.9)	2 (3.8)	NA	NA	NA	NA		
Not PPV or PCV serotypes	16 (21.6)	6 (11.3)	NA	NA	NA	NA		
NA	19 (25.7)	44 (83.0)	NA	NA	NA	NA		
**Antimicrobial resistance (n, %)**								
Penicillin	NA	NA	10 (12.7)	5 (4.7)	NA	NA		
Erythromycin	NA	NA	12 (17.1)	11 (16.9)	NA	NA		
Cefotaxime/Ceftriaxone	NA	NA	2 (1.9)	2 (1.4)	NA	NA		
